# Targeting ACE2-BRD4 crosstalk in colorectal cancer and the deregulation of DNA repair and apoptosis

**DOI:** 10.1038/s41698-023-00361-4

**Published:** 2023-02-18

**Authors:** Shilan Zhang, Sabeeta Kapoor, Chakrapani Tripathi, Jorge Tovar Perez, Nivedhitha Mohan, Wan Mohaiza Dashwood, Ke Zhang, Praveen Rajendran, Roderick Dashwood

**Affiliations:** 1 Center for Epigenetics & Disease Prevention, Texas A&M Health, and Department of Translational Medical Sciences, Texas A&M School of Medicine, Houston, TX USA; 2 Department of Clinical Cancer Prevention, The University of Texas MD Anderson Cancer Center, Houston, TX USA

**Keywords:** Colon cancer, Molecular medicine

## Abstract

ACE2 overexpression in colorectal cancer patients might increase susceptibility to SARS-CoV-2 infection. We report that knockdown, forced overexpression, and pharmacologic inhibition in human colon cancer cells targeted ACE2-BRD4 crosstalk to mediate marked changes in DNA damage/repair and apoptosis. In colorectal cancer patients for whom high *ACE2* plus high *BRD4* expression is predictive of poor survival, pan-BET inhibition would need to consider proviral/antiviral actions of different BET proteins during SARS-CoV-2 infection.

A recent investigation reported on the high expression of angiotensin I-converting enzyme 2 (ACE2) and clinical characteristics of Coronavirus Disease 2019 (COVID-19) in colorectal cancer patients^1^. COVID-19 patients often present with gastrointestinal maladies^2^, and severe acute respiratory syndrome coronavirus 2 (SARS-CoV-2) RNA can persist in fecal samples from affected individuals^3^. As a key entry receptor for SARS-CoV-2 into human cells, *ACE2* was expressed at high levels on tumor and normal colorectal epithelial tissues^1^. Liu et al. concluded that patients with colorectal cancer may be particularly susceptible to viral infection^1^.

To circumvent *ACE2* overexpression and viral entry into cells one approach might involve the repurposing of bromodomain and extraterminal domain (BET) inhibitors, which are known to effectively downregulate *MYC* and other oncogenic targets in cancer cells^4–7^. Recent reports, however, have raised questions regarding this potential therapeutic strategy. In lung cancer cells, viral replication was exacerbated after chemical or genetic inactivation of BET proteins^8^, and high *ACE2* expression in the tumors from colorectal cancer patients was synonymous with better survival^9^. To clarify the underlying mechanisms, we examined phenotypic and mechanistic readouts following ACE2 knockdown and forced overexpression in human colon cancer cells, as well as pharmacologic BET inhibition.

We first corroborated the high ACE2 protein expression in human colorectal cancers (Fig. 1a, b), which in prior studies was synonymous with better patient survival (*p* = 0.028)^9^. Because in lung cancer cells BRD2 was considered “proviral” and positively regulated expression of the ACE2 receptor whereas BRD4 was “antiviral”^8^, these BET proteins were examined in colorectal cancer datasets. For BRD2, low-to-moderate immunopositivity in adenocarcinomas was associated with a non-significant difference in survival (*p* = 0.11, https://www.proteinatlas.org/ENSG00000204256-BRD2/pathology/colorectal+cancer). In contrast, BRD4 was overexpressed in colorectal cancers (Fig. 1c, d) and predicted significantly reduced survival (https://www.proteinatlas.org/ENSG00000141867-BRD4/pathology/colorectal+cancer, *p* = 0.0064). Moreover, high expression of both *ACE2* and *BRD4* mRNAs compared with low *ACE2* plus low *BRD4* levels also predicted significantly reduced survival in colorectal cancer patients (*p* = 0.0174, Fig. 1e).

**Fig. 1:**
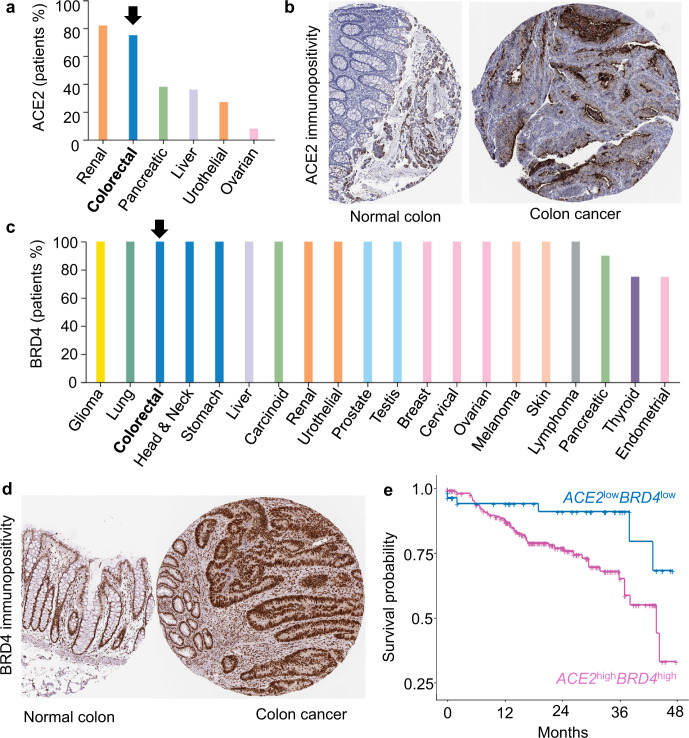
Overexpression of ACE2 and BRD4 in human colorectal cancer and associated survival outcomes. **a** ACE2 expression in human malignancies via immunohistochemical (IHC) analysis. **b** Representative tissue microarrays (TMAs) of ACE2 expression in colorectal adenocarcinoma vs. normal colon. **c** BRD4 protein expression in human malignancies via IHC. **d** Representative TMAs of BRD4 protein expression in colorectal adenocarcinoma vs. normal colon. **e** Kaplan–Meier survival curves for colorectal cancer patients in which tumors exhibited high *ACE2* plus high *BRD4* mRNA levels vs. low *ACE2* plus low *BRD4* mRNA expression (*p* = 0.0174). In **a**–**d**, information was obtained from The Human Protein Atlas (https://www.proteinatlas.org/), whereas **e** was generated after data mining of The Cancer Genome Atlas (TCGA) (https://www.genome.gov/Funded-Programs-Projects/Cancer-Genome-Atlas), as reported^9,19^.

Extending our prior immunoblotting work in cell-based assays^6,7,10–12^, screening of SW48, CaCo2, HT29, LoVo and SW620 human colon cancer cell lines revealed moderate to high constitutive expression of ACE2, BRD2 and BRD4 proteins compared with CCD841 normal colonic epithelial cells (Supplementary Fig. 1). In pilot studies, commercially-available shRNA shACE2-1 diminished expression of the ACE2 target protein in SW480 and SW620 cells coincident with (1) increased DNA damage and repair makers^10–12^, such as pH2AX, (2) reduced cell viability and colony formation, and (3) enhanced apoptosis (Supplementary Figs. 2 and 3). Under the same experimental conditions, ACE2 knockdown using shACE2-2 also diminished cell viability and colony formation, but in the metastasis-associated SW620 cell lineage shACE2-2 was less effective in terms of pH2AX and apoptosis induction (Supplementary Fig. 3a, d). Additional shRNA hairpins, pooled siRNAs, or guide RNAs might achieve enhanced ACE2 knockdown and associated phenotypic readouts, with careful attention to potential off-target effects in each scenario. In the present study, the more effective shACE2-1 was carried forward as “shACE2” for follow-up experiments in SW480 and SW620 cells, which revealed markedly reduced BRD4 and c-Myc protein expression compared with scrambled shRNA controls, and a concomitant increase in pH2AX and pRPA32 (Fig. 2a). Morphological examination and quantification in the CCK8 assay revealed that ACE2 knockdown reduced cell viability significantly (Fig. 2b), and the colony-formation assay confirmed a decrease in the number of crystal-violet-stained cells (Fig. 2c). On the other hand, fluorescence-activated cell sorting (FACS) analyses identified a significant increase in the percentage of apoptotic cells following ACE2 knockdown (Fig. 2d).

**Fig. 2:**
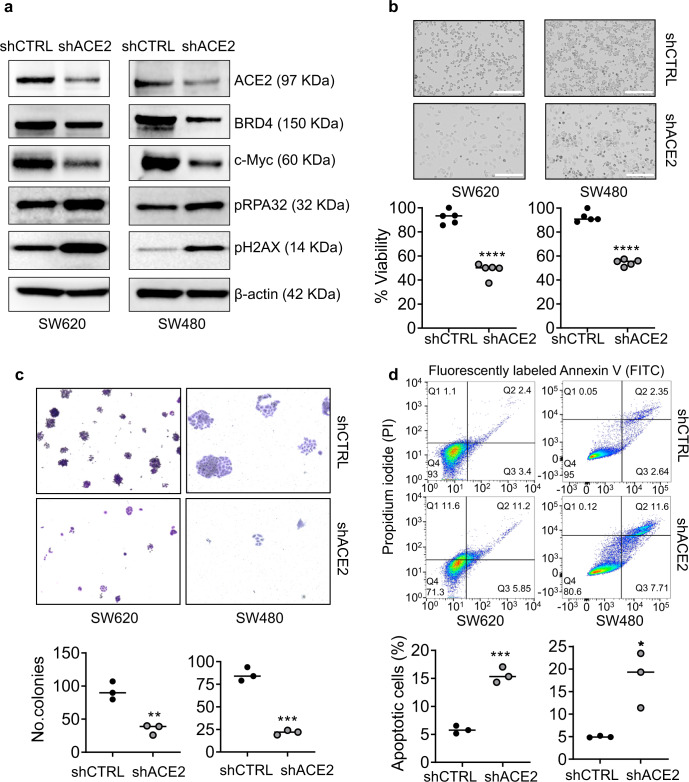
ACE2 regulates BRD4 expression, DNA repair and apoptosis in human colon cancer cells. **a** Immunoblotting in SW620 and SW480 cells 48 h after scrambled shRNA control (shCTRL) or ACE2 shRNA treatment (shACE2-1 from Supplementary Figs. 2 and 3), with β-actin as loading control. **b** Morphology and cell viability in the CCK8 assay; scale bar = 200 μm. **c** Representative images (×4 magnification) from the colony-formation assay and quantification of crystal-violet-stained colonies. **d** Fluorescence-activated cell sorting (FACS) and quantification of apoptosis 48 h after ACE2 knockdown. Difference between means for *n* = 5 or *n* = 3 replicates, as indicated; **p < 0.01, ***p < 0.001, ****p < 0.0001 by Student’s *t* test vs. shCTRL. In GraphPad Prism 9.4.1.

Next, experiments employed transient transfection of an expression construct for Myc-tagged ACE2, which revealed enhanced BRD4 levels, whereas c-Myc remained at a more-or-less constant high level in SW620 cells (Fig. 3a). Normalizing to β-actin loading controls from replicate immunoblotting experiments revealed a positive correlation between BRD4 and ACE2 in SW620 cells (Fig. 3b). Similar observations were made in other cell lines, such as SW480 cells, at doses of transfected Myc-tagged ACE2 protein tested below the threshold for cell detachment and cell rounding (Supplementary Fig. 4). Increasing levels of transfected Myc-tagged ACE2 enhanced cell viability and migration endpoints significantly (Fig. 3c, d), reversing phenotypes observed in the ACE2 knockdown experiments (Fig. 2).

**Fig. 3:**
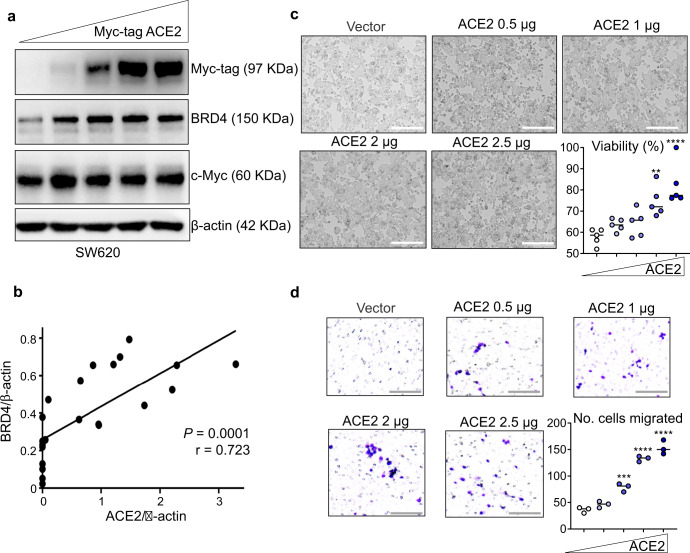
Forced expression of ACE2 increases endogenous BRD4 and enhances the viability and migration of colon cancer cells. **a** Immunoblotting 48 h after transient transfection of 0 (vector alone), 0.5, 1, 2, or 2.5 µg Myc-tagged ACE2 in SW620 cells. **b** Densitometric analyses in SW620 cells of BRD4 vs. ACE2, normalized to β-actin, after replicate experiments (as in **a**). **c** Representative images and cell viability data in the CCK8 assay; scale bars = 200 μm. **d** Representative images and quantification of SW620 cell migration after crystal violet-staining (scale bars = 200 μm). In **c**, **d**, difference between means from *n* = 5 or *n* = 3 replicates, as indicated, 48 h after transient transfection of Myc-tagged ACE2, as in **a** (wedge symbol); **p < 0.01, ***p < 0.001, ****p < 0.0001 by Student’s *t* test vs. 0-µg vector control. GraphPad Prism 9.4.1.

Attention next was focused on pharmacologic inhibition in human colon cancer cells. Downregulation of ACE2 and c-Myc protein expression was observed in pilot experiments with the BET inhibitor JQ1 and the BET degraders MZ1 and dBET6 (Supplementary Fig. 5). The latter test agent was shown previously to preferentially target the loss of BRD4 over BRD2 and other BET family members in human colon cancer cells^7^, and was taken into additional experiments. Following treatment with 0.5–3 μM dBET6, concentration-dependent inhibition of cell viability and colony formation (Fig. 4a, b) and a concomitant increase in the percentage of apoptotic SW620 cells was observed (Fig. 4c). Immunoblotting of the cell lysates revealed that with the loss of BRD4 expression after dBET6 treatment, ACE2 and c-Myc protein levels were reduced markedly, whereas increased cleaved poly(ADP-ribose)polymerase (PARP), pH2AX and pRPA32 were indicative of enhanced apoptosis and DNA damage/repair activities (Fig. 4d). Transiently transfected ACE2 dose-dependently reversed the PARP cleavage associated with loss of BRD4 upon dBET6 treatment (Fig. 4e). Additional rescue experiments were not conducted on cell migration and viability phenotypes, which represents a limitation of the current study worthy of further investigation. Finally, we reported previously on the tumor growth inhibition and loss of BRD4 protein expression in SW620 cells grown as xenografts in nude mice treated with dBET6, alone or in combination with a histone deacetylase inhibitor^7^. The corresponding biobanked SW620 cell lysates revealed marked depletion of ACE2 in mice treated with dBET6 in vivo (Fig. 4f, red box).

**Fig. 4:**
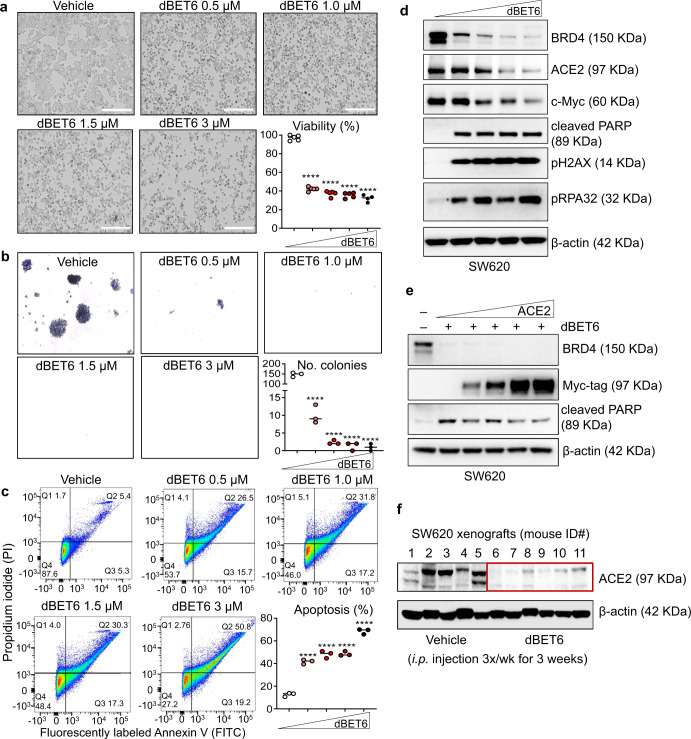
Pharmacologic inhibition of BRD4 depletes endogenous ACE2 in colon cancer cells while reducing cell viability and increasing apoptosis. **a** Representative images of SW620 cells 48 h after treatment with BRD4 antagonist dBET6; scale bar = 200 μm. The associated cell viability data were obtained from the CCK8 assay at 48 h. **b** Representative images and quantification from the colony-formation assay at 48 h. **c** Apoptosis quantified at 48 h by FACS analysis. **d** Cell lysates from panel a immunoblotted for target proteins; wedge symbol = 0, 0.5, 1, 1.5 and 3 μM dBET6. **e** Immunoblotting of SW620 cells mock-transfected (–) or transiently transfected with Myc-tagged ACE2 ± 1 µM dBET6 for 48 h. Wedge symbol = 0, 0.5, 1, 2 and 3 μg Myc-tagged ACE2. **f** Nude mice received vehicle or dBET6 at a dose of 7.5 mg/kg body weight by i.p. injection, 3 times per week for 3 weeks (*n* = 5–6 animals/group). Tumor growth inhibition and molecular target modulation, including BRD4 protein loss, were reported previously^7^. The biobanked SW620 cell lysates exhibited reduced ACE2 protein expression after dBET6 treatment in vivo (red box). In **a**–**c**, difference between means from *n* = 3 replicates; ****p < 0.0001 by Student’s *t* test vs. vehicle control. GraphPad Prism 9.4.1.

Collectively, the results from this study and prior reports^8,9^ highlight the yin/yang nature of targeting ACE2 in cancer etiology. Knockdown and overexpression experiments in human colon cancer cells implicated an apparent oncogenic role for ACE2, with pharmacologic BET inhibition recapitulating molecular and phenotypic readouts, including loss of c-Myc, cell growth inhibition, and apoptosis induction. Future work should clarify the precise mechanisms of ACE2-BRD4 crosstalk, acting at the level of transcriptional regulation, post-translational modifications and/or protein-protein interactions^4–7,10–17^. This could include preclinical models in which Ace2 overexpression is observed in early adenomas relative to adjacent normal-looking colonic mucosa (Supplementary Fig. 6). Precision oncology approaches might, in principle, target colorectal cancers in which high *ACE2* plus high *BRD4* expression is predictive of poor survival (Fig. 1e). In lung epithelial cells, transcriptional repression with BET antagonists inhibited SARS-CoV-2 infection in vitro^18^. However, Chen et al.^8^ noted that most BET inhibitors do not distinguish between different BET proteins, and urged caution in the therapeutic use of pan-BET inhibitors in people at risk for COVID-19, concluding that “a molecule that specifically inhibits BRD2 but not BRD4 could be useful”. Risk-benefit of prior vs. concurrent BET inhibitor treatment in colorectal cancer patients with SARS-CoV-2 infection warrants further investigation.

## Methods

### Cell-based assays

Human colon cancer cell lines (SW48, SW480, SW620, Caco2, HT29, LoVo) and non-transformed colonic epithelial cells (CCD841) were obtained from ATCC and validated as reported^7,17^. Chemicals, reagents, and cell culture media were purchased from Invitrogen, dBET6, JQ1 and MZ1 from MedChemExpress, ACE2 shRNA plasmids (#sc-41400-SH) from Santa Cruz Biotechnology, and ACE2 overexpression plasmid with an N-terminal Myc tag (#141185) from Addgene. Plating-densities and cell culture conditions followed the corresponding methodologies for CCK8 cell viability assays, colony formation, cell migration, and FACS-based assessment of apoptosis^6,7^, as briefly described below.

#### Cell viability

Colon cancer cells in exponential growth phase were plated at 5000 cells per well in 96-well tissue culture plates. After attachment overnight, cells were treated for 48 h in triplicate wells and assessed for viability using Cell Counting Kit-8 (CCK8, APExBIO, Boston, MA, USA).

#### Colony formation

Colon cancer cells were trypsinized and plated in 6-well dishes (500 cells/well), allowed to attach overnight, and then treated for 48 h in triplicate wells. Seven days later, colonies were fixed, stained with crystal violet, and counted. The surviving fraction was calculated as the ratio of the number of colonies in the treated sample to the number of colonies in the control.

#### Cell migration

Colon cancer cells were added to the upper chamber of a transwell with an 8-µm filter, and McCoy’s medium supplemented with 20% FBS was added to the lower chamber in triplicate wells. After 24 h, cells that migrated to the lower side of the filter were fixed and stained with crystal violet, followed by counting three random fields under an inverted microscope.

#### Apoptosis

Colon cancer cells were treated for 48 h and apoptosis assays were conducted in triplicate using a BD Pharmingen PE Annexin V Apoptosis Detection Kit I (BD Biosciences, San Jose, CA, USA). Briefly, cells were collected, washed with PBS, and incubated in binding buffer with 5 μl of PE Annexin V for 5 min and 5 μl of 7-AAD for 15 min in the dark at 37 °C. Percent of apoptotic cells was determined using LSR II Flow cytometer (BD Biosciences) and FlowJo 10.8.1 software. For the FACS gating strategy, refer to Supplementary Fig. 7.

#### Overexpression and knockdown

Colon cancer cells at 80% confluency were transfected in triplicate either with 2.5 µg of plasmid DNA using Lipofectamine 3000 transfection reagent (Invitrogen) or with 10 µM of shRNA plasmid using Lipofectamine RNAiMAX reagent (Invitrogen), followed by 48 h incubation at 37 °C.

### Immunoblotting

Immunoblotting used published procedures for whole cell lysates^6,7,10^. In brief, proteins (20 µg/lane) were separated by SDS-PAGE on NuPAGE 4–12% Bis-Tris gels (Invitrogen) and transferred to nitrocellulose membranes. Membranes were saturated with 2% BSA for 1 h, followed by overnight incubation at 4 °C with primary antibodies for BRD4 (1:1000, #E2A7X), c-Myc (1:1000, #D3N8F), PARP (1:1000, #9542) and ACE2 (1:1000, #4355) from Cell Signaling (Danvers), pH2AX Ser139 (Santa Cruz, 1:1000, #101696), pRPA32 S4/S8 (Bethyl Laboratories, 1:500, #A300-245A), and β-actin (Sigma, 1:3000, #A5441). After washing, membranes were incubated with horseradish peroxidase-conjugated secondary antibodies for 1 h. Bands were visualized using Western Lightning Plus-ECL Enhanced Chemiluminescence Substrate (Perkin Elmer) and detected using a ChemiDoc MP Imaging System (Bio-Rad), based on three or more replicate experiments. For uncropped images with the molecular weight marker ladder, refer to Supplementary figures.

### Preclinical

Xenograft experiments received prior approval from the Institutional Animal Care and Use Committee, and were performed on *n* = 5–6 animals/group^7^. In brief, 5 × 10^6^ SW620 cells were injected into either flank of male athymic nude mice (Envigo). After a week, animals were treated with vehicle or 7.5 mg/kg body weight of dBET6, thrice per week for 3 weeks by i.p. injection. Tumor volumes were measured twice per week using Vernier calipers, and the corresponding tumors were examined at the end of the study for molecular target modulation, as reported^7^. In brief, xenografts were lysed using IP lysis buffer containing protease inhibitors^7^, followed by immunoblotting of target proteins, as described above.

### Statistics

Results are representative of the findings from three or more biological and technical replicates, expressed as mean ± SD, as reported^6,19,20^. Paired group comparisons were made for treatment vs. control using Student’s *t* test in GraphPad Prism 9. The Cancer Genome Atlas (TCGA) was mined for survival analysis using R programming language and the log-rank test was conducted to compare Kaplan–Meier curves, as reported^9,21^. Statistical significance was indicated in the figures as follows: **p < 0.01, ***p < 0.001, and ****p < 0.0001.

### Reporting summary

Further information on research design is available in the Nature Research Reporting Summary linked to this article.

## Supplementary information

Supplementary Info

REPORTING SUMMARY

## Data Availability

Data generated during this investigation are available from the corresponding authors upon reasonable request. CRC survival data and transcript read counts were obtained from TCGA (https://portal.gdc.cancer.gov). Additional information was obtained from The Human Protein Atlas at: https://www.proteinatlas.org/ENSG00000130234-ACE2.
