# Supplementary material for: Targeting ACE2-BRD4 crosstalk in colorectal cancer and the deregulation of DNA repair and apoptosis
Source: NPJ Precis Oncol. 2023 Feb 18;7:20. doi: 10.1038/s41698-023-00361-4 (PMC9938505; doi:10.1038/s41698-023-00361-4)
Supplement: Supplementary file 1 — Supplementary Info [file 41698_2023_361_MOESM1_ESM.pdf]

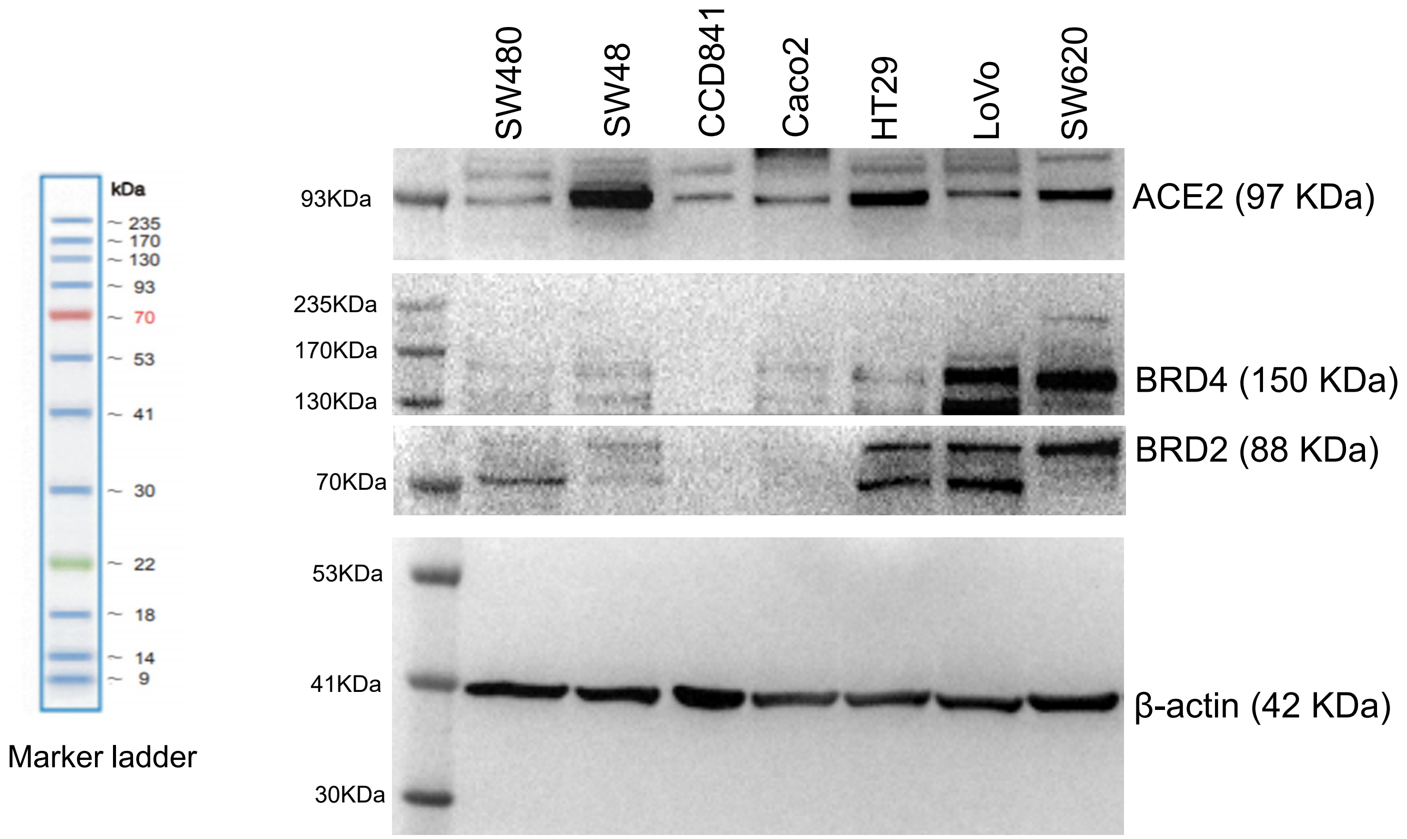

Supplementary Fig 1. Constitutive expression of ACE2 in human colon cancer cells. Immunoblotting of ACE2 and BET proteins in a panel of human colon cancer cell lines and in CCD841 normal colonic epithelial cells. β-actin, loading control.

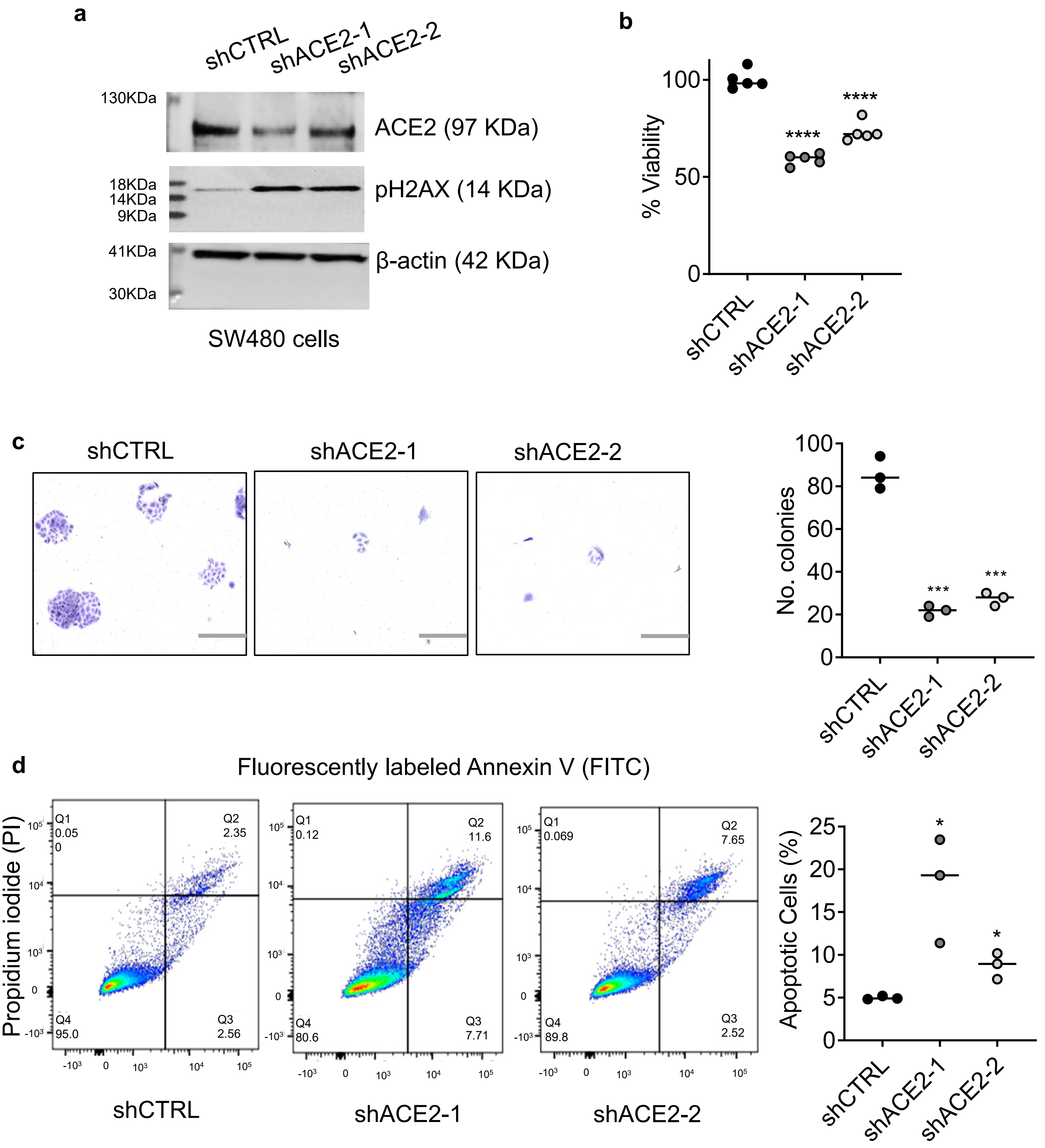

Supplementary Fig 2 ACE2 regulates DNA repair, cell viability and apoptosis in SW480 human colon cancer cells. **a** Immunoblotting 48 h after treatment of SW480 cells with scrambled shRNA control (shCTRL) or two different small hairpin RNAs targeting ACE2 (shACE2-1, shACE2-2), with β-actin as loading control. **b** Cell viability in the CCK8 assay. **c** Representative images (4x magnification) from the colony formation assay and quantification of crystal-violet-stained colonies; scale bar = 200 µm. **d** Fluorescence-activated cell sorting (FACS) and quantification of apoptosis 48 h after ACE2 knockdown. Difference between means for n=5 or n=3 replicates, as indicated; *P <0.05, ***P <0.001, ****P <0.0001 by Student's *t*-test *vs.* shCTRL in GraphPad Prism 9.4.1

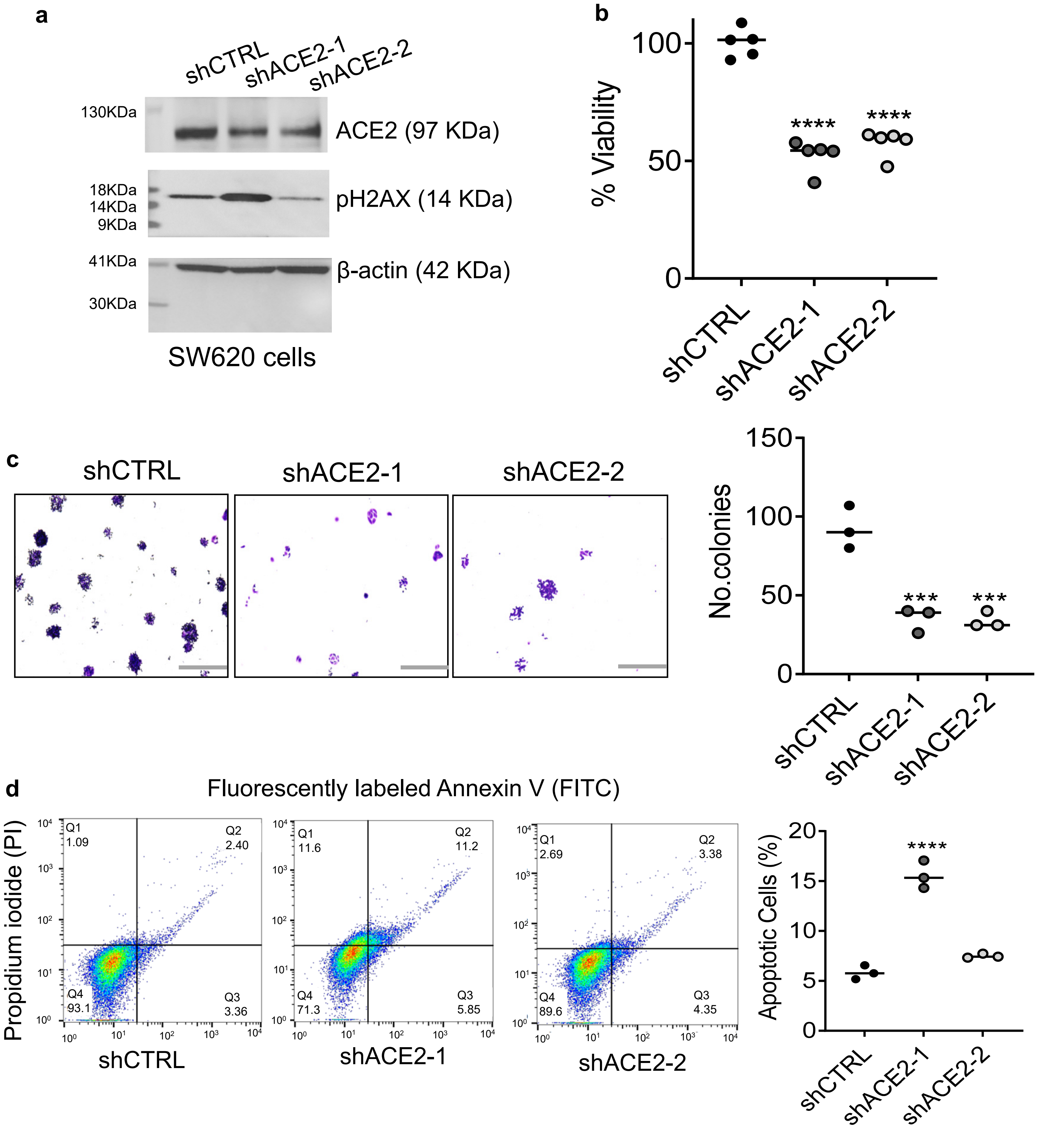

Supplementary Fig 3 ACE2 regulates DNA repair, cell viability and apoptosis in SW620 human colon cancer cells. **a** Immunoblotting 48 hours after treatment of SW620 cells with scrambled shRNA control (shCTRL) or two different small hairpin RNAs targeting ACE2 (shACE2-1, shACE2-2), with β-actin as loading control. **b** Cell viability in the CCK8 assay. **c** Representative images (4x magnification) from the colony formation assay and quantification of crystal-violet-stained colonies. scale bar = 200 μm. **d** Fluorescence-activated cell sorting (FACS) and quantification of apoptosis 48 h after ACE2 knockdown. Difference between means for n=5 or n=3 replicates, as indicated; ****P* <0.001, *****P* <0.0001 by Student's *t*-test *vs.* shCTRL in GraphPad Prism 9.4.1

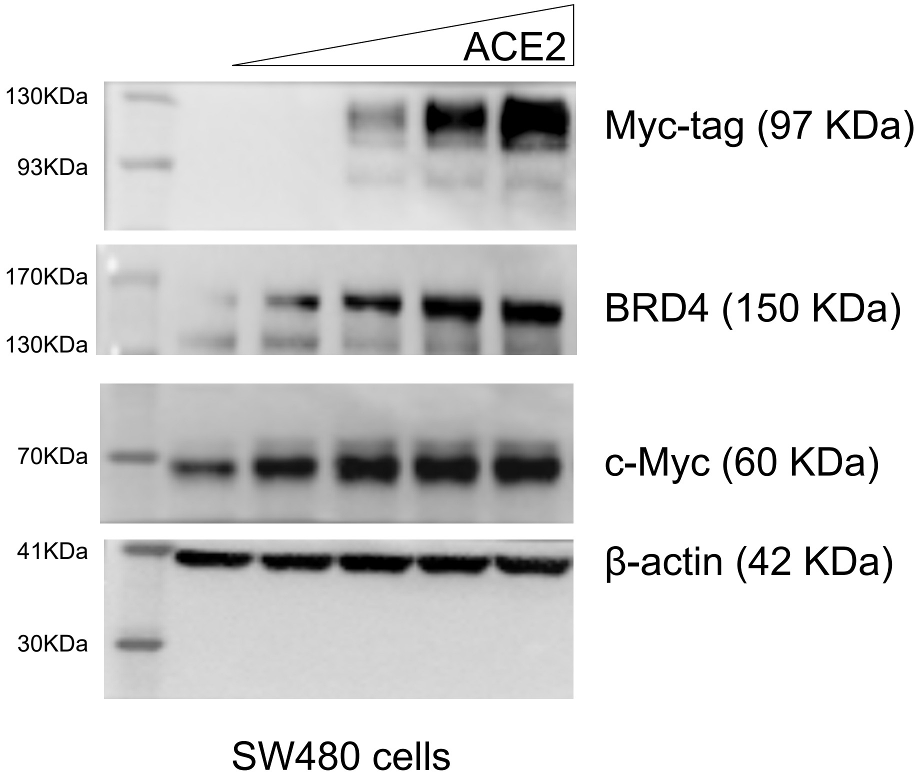

Supplementary Fig 4. Forced expression of ACE2 increases endogenous BRD4 and c-Myc in SW480 colon cancer cells. Immunoblotting 48 hours after transient transfection of 0 (vector alone), 0.5, 1, or 2 µg Myc-tagged ACE2. In SW480 cells, higher doses of transfected Myc-tagged ACE2 were associated with increased cell detachment and cell rounding at 48 hours, indicative of toxicity (data not shown). β-actin, loading control.

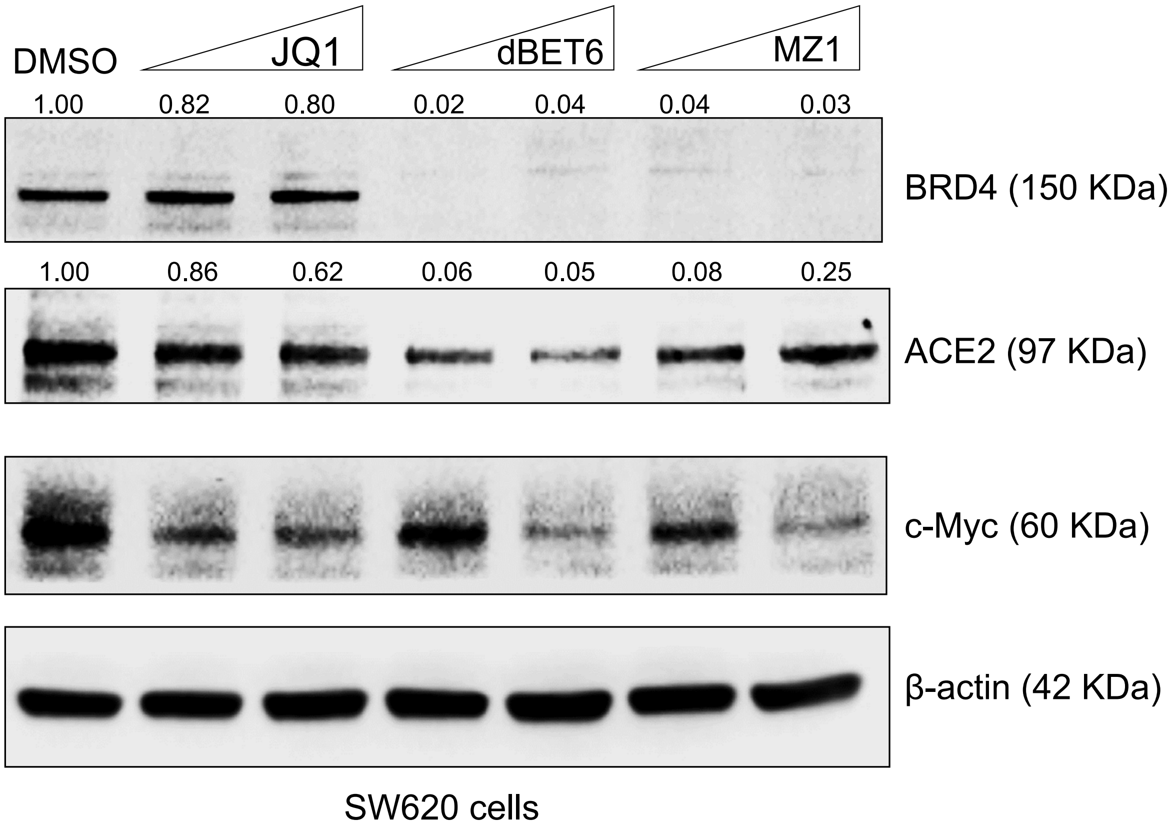

Supplementary Fig 5. BET inhibitors and degraders lower endogenous ACE2 and c-Myc expression in human colon cancer cells. Immunoblotting of SW620 cells 48 hours after treatment with BET inhibitor JQ1 (0.5, 1 μM) and the PROTAC BET degraders dBET6 (1.5, 3 μM), and MZ1 (0.5, 1 μM). β-actin, loading control. Following densitometric analysis, BRD4:β-actin and ACE2:β-actin ratios were determined relative to vehicle control, which was assigned an arbitrary value of 1.00.

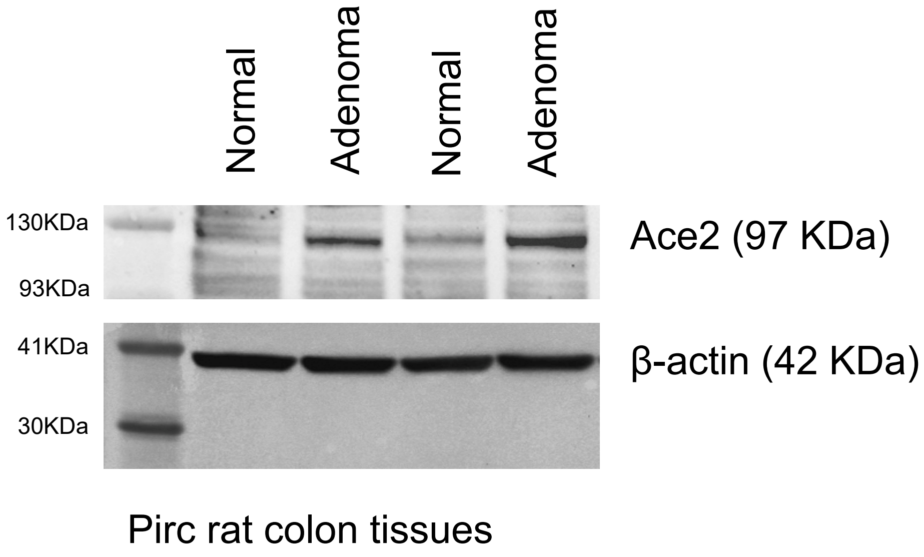

Supplementary Fig 6. ACE2 overexpression in rat colon adenomas. Immunoblotting of ACE2 in colon adenomas and adjacent normal-looking colonic mucosa in the Apc-mutant polyposis in rat colon (Pirc) model[6], with β-actin as loading control.

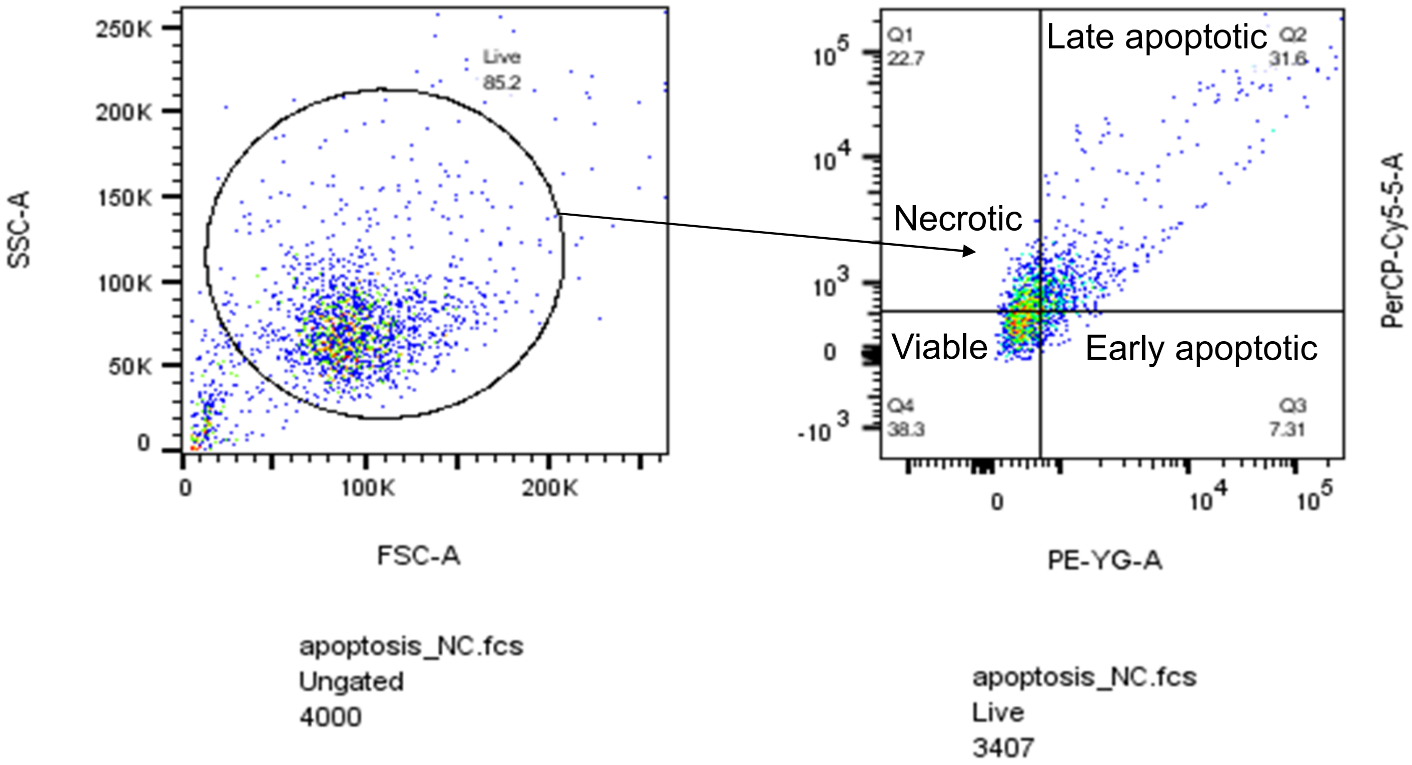

Supplementary Fig 7. Gating strategies for cell sorting via FACS. Gating sorted Propidium iodide/Annexin V-stained apoptotic colon cancer cells, as presented in Figs 2D and 4C. Cells in the different quadrants represent the corresponding apoptotic status, as follows: Q1 (necrotic), Q2 (late apoptotic), Q3 (early apoptotic), and Q4 (viable, non-apoptotic).
