# Supplementary material for: Targeting ACE2-BRD4 crosstalk in colorectal cancer and the deregulation of DNA repair and apoptosis
Source: NPJ Precis Oncol. 2023 Feb 18;7:20. doi: 10.1038/s41698-023-00361-4 (PMC9938505; doi:10.1038/s41698-023-00361-4)
Supplement: Supplementary file 2 — REPORTING SUMMARY [file 41698_2023_361_MOESM2_ESM.pdf]

nature portfolio

Corresponding author(s): Professor Roderick Dashwood

Last updated by author(s): Oct 28, 2022

Reporting Summary

Nature Portfolio wishes to improve the reproducibility of the work that we publish. This form provides structure for consistency and transparency in reporting. For further information on Nature Portfolio policies, see our Editorial Policies and the Editorial Policy Checklist.

Statistics

For all statistical analyses, confirm that the following items are present in the figure legend, table legend, main text, or Methods section.

n/a	Confirmed	
☐	☒	The exact sample size (*n*) for each experimental group/condition, given as a discrete number and unit of measurement
☐	☒	A statement on whether measurements were taken from distinct samples or whether the same sample was measured repeatedly
☐	☒	The statistical test(s) used AND whether they are one- or two-sided<br>*Only common tests should be described solely by name; describe more complex techniques in the Methods section.*
☒	☐	A description of all covariates tested
☒	☐	A description of any assumptions or corrections, such as tests of normality and adjustment for multiple comparisons
☒	☐	A full description of the statistical parameters including central tendency (e.g. means) or other basic estimates (e.g. regression coefficient) AND variation (e.g. standard deviation) or associated estimates of uncertainty (e.g. confidence intervals)
☒	☐	For null hypothesis testing, the test statistic (e.g. *F*, *t*, *r*) with confidence intervals, effect sizes, degrees of freedom and *P* value noted<br>*Give P values as exact values whenever suitable.*
☒	☐	For Bayesian analysis, information on the choice of priors and Markov chain Monte Carlo settings
☒	☐	For hierarchical and complex designs, identification of the appropriate level for tests and full reporting of outcomes
☒	☐	Estimates of effect sizes (e.g. Cohen's *d*, Pearson's *r*), indicating how they were calculated

Our web collection on statistics for biologists contains articles on many of the points above.

Software and code

Policy information about availability of computer code

Data collection	Human Protein Atlas, The Cancer Genome Atlas (TCGA), GraphPad Prism 9
Data analysis	N/A

For manuscripts utilizing custom algorithms or software that are central to the research but not yet described in published literature, software must be made available to editors and reviewers. We strongly encourage code deposition in a community repository (e.g. GitHub). See the Nature Portfolio guidelines for submitting code & software for further information.

Data

Policy information about availability of data

All manuscripts must include a data availability statement. This statement should provide the following information, where applicable:

- Accession codes, unique identifiers, or web links for publicly available datasets
- A description of any restrictions on data availability
- For clinical datasets or third party data, please ensure that the statement adheres to our policy

Data generated during this investigation are available from the corresponding authors upon reasonable request. The CRC survival data and transcript read counts were obtained from the TCGA data portal (https://portal.gdc.cancer.gov). Additional information was obtained from The Human Protein Atlas at URL: https://www.proteinatlas.org/ENSG00000130234-ACE2

Human research participants

Policy information about studies involving human research participants and Sex and Gender in Research.

Reporting on sex and gender	N/A
Population characteristics	N/A
Recruitment	N/A
Ethics oversight	N/A

Note that full information on the approval of the study protocol must also be provided in the manuscript.

Field-specific reporting

Please select the one below that is the best fit for your research. If you are not sure, read the appropriate sections before making your selection.

☒ Life sciences ☐ Behavioural & social sciences ☐ Ecological, evolutionary & environmental sciences

For a reference copy of the document with all sections, see nature.com/documents/nr-reporting-summary-flat.pdf

Life sciences study design

All studies must disclose on these points even when the disclosure is negative.

Sample size	Results are representative of the findings from three or more biological and technical replicates, expressed as mean±SD. Paired group comparisons were made for treatment vs. control using Student's t-test in GraphPad Prism 9. Statistical significance was indicated in the figures as follows: **P <0.01, ***P <0.001, and ****P <0.0001.
Data exclusions	No data were excluded.
Replication	All experiments were reproduced to reliably support conclusions stated in the manuscript.
Randomization	NA
Blinding	Key measurements was carried out in a blinded manner.

Reporting for specific materials, systems and methods

We require information from authors about some types of materials, experimental systems and methods used in many studies. Here, indicate whether each material, system or method listed is relevant to your study. If you are not sure if a list item applies to your research, read the appropriate section before selecting a response.

Materials & experimental systems

n/a	Involved in the study	
☐	☒	Antibodies
☐	☒	Eukaryotic cell lines
☐	☐	Palaeontology and archaeology
☐	☒	Animals and other organisms
☐	☐	Clinical data
☐	☐	Dual use research of concern

Methods

n/a	Involved in the study	
☐	☐	ChIP-seq
☐	☒	Flow cytometry
☐	☐	MRI-based neuroimaging

Antibodies

Antibodies used	BRD4 (#E2A7X), c-Myc (#D3N8F), PARP (#9542) and ACE2 (#4355) from Cell Signaling (Danvers), pH2AX Ser139 (Santa Cruz, #101696), pRPA32 S4/S8 (Bethyl Laboratories, #A300-245A), and β-actin (Sigma, #A5441).
Validation	Antibodies were validated by the manufacturer, and we tested them on cell/tissue lysates at the recommended experimental conditions by immunoblotting. Additionally, knockdown and over-expression experiments validated ACE2 antibodies, the main subject of the manuscript.

Eukaryotic cell lines

Policy information about cell lines and Sex and Gender in Research

Cell line source(s)	Cell lines were purchased from ATCC and used within 10–15 passages from receipt. Human colon cancer cells (SW480, SW620) is male and non-transformed colonic epithelial cell line (CCD841) is female.
Authentication	All cell lines authenticated by Idexx Radil, Columbia, MO, USA.
Mycoplasma contamination	All cell lines tested negative for mycoplasma contamination.
Commonly misidentified lines (See ICLAC register)	None of the used cell lines is listed in ICLAC database

Palaeontology and Archaeology

Specimen provenance	NA
Specimen deposition	NA
Dating methods	NA

☐ Tick this box to confirm that the raw and calibrated dates are available in the paper or in Supplementary Information.

Ethics oversight	NA

Note that full information on the approval of the study protocol must also be provided in the manuscript.

Animals and other research organisms

Policy information about studies involving animals; ARRIVE guidelines recommended for reporting animal research, and Sex and Gender in Research

Laboratory animals	No new animal studies were conducted. Rodent tissues from mice/rats were from previously reported studies.
Wild animals	NA
Reporting on sex	NA
Field-collected samples	NA
Ethics oversight	TAMU IACUC

Note that full information on the approval of the study protocol must also be provided in the manuscript.

Clinical data

Policy information about clinical studies

All manuscripts should comply with the ICMJE guidelines for publication of clinical research and a completed CONSORT checklist must be included with all submissions.

Clinical trial registration	NA
Study protocol	NA
Data collection	NA
Outcomes	NA

Dual use research of concern

Policy information about dual use research of concern

Hazards

Could the accidental, deliberate or reckless misuse of agents or technologies generated in the work, or the application of information presented in the manuscript, pose a threat to:

No	Yes	
☒	☐	Public health
☒	☐	National security
☒	☐	Crops and/or livestock
☒	☐	Ecosystems
☒	☐	Any other significant area

Experiments of concern

Does the work involve any of these experiments of concern:

No	Yes	
☒	☐	Demonstrate how to render a vaccine ineffective
☒	☐	Confer resistance to therapeutically useful antibiotics or antiviral agents
☒	☐	Enhance the virulence of a pathogen or render a nonpathogen virulent
☒	☐	Increase transmissibility of a pathogen
☒	☐	Alter the host range of a pathogen
☒	☐	Enable evasion of diagnostic/detection modalities
☒	☐	Enable the weaponization of a biological agent or toxin
☒	☐	Any other potentially harmful combination of experiments and agents

ChIP-seq

Data deposition

☐ Confirm that both raw and final processed data have been deposited in a public database such as GEO.

☐ Confirm that you have deposited or provided access to graph files (e.g. BED files) for the called peaks.

Data access links<br>*May remain private before publication.*	NA
Files in database submission	NA
Genome browser session<br>(e.g. UCSC)	NA

Methodology

Replicates	NA
Sequencing depth	NA
Antibodies	NA
Peak calling parameters	NA
Data quality	NA
Software	NA

Flow Cytometry

Plots

Confirm that:

☒ The axis labels state the marker and fluorochrome used (e.g. CD4-FITC).

☒ The axis scales are clearly visible. Include numbers along axes only for bottom left plot of group (a 'group' is an analysis of identical markers).

☒ All plots are contour plots with outliers or pseudocolor plots.

☒ A numerical value for number of cells or percentage (with statistics) is provided.

Methodology

Sample preparation	Colon cancer cells were treated with test compounds for 72 h in 6-well plates, and apoptosis assays were conducted using a BD Pharmingen PE Annexin V Apoptosis Detection Kit I (BD Biosciences, San Jose, CA, USA).
Instrument	LSR II Flow cytometer (BD Biosciences)
Software	FlowJo 10.8.1 software.
Cell population abundance	cell sorting not employed
Gating strategy	Using the FSC/SSC gating, debris was removed by gating on the untreated control cell population. Identical positivity threshold was applied to all samples within a cell line.

☒ Tick this box to confirm that a figure exemplifying the gating strategy is provided in the Supplementary Information.

Magnetic resonance imaging

Experimental design

Design type	NA
Design specifications	NA
Behavioral performance measures	NA

Acquisition

Imaging type(s)	NA
Field strength	NA
Sequence & imaging parameters	NA
Area of acquisition	NA

Diffusion MRI ☐ Used ☒ Not used

Preprocessing

Preprocessing software	NA
Normalization	NA
Normalization template	NA
Noise and artifact removal	NA
Volume censoring	NA

Statistical modeling & inference

Model type and settings	Two-group comparison with Student's t test and survival analysis using log-rank test.
Effect(s) tested	Protein levels, cell viability, patient overall survival

Specify type of analysis: ☐ Whole brain ☐ ROI-based ☐ Both

Statistic type for inference (See Eklund et al. 2016)	*Specify voxel-wise or cluster-wise and report all relevant parameters for cluster-wise methods.*
Correction	*Describe the type of correction and how it is obtained for multiple comparisons (e.g. FWE, FDR, permutation or Monte Carlo).*

Models & analysis

n/a	Involved in the study
☒	☐ Functional and/or effective connectivity
☒	☐ Graph analysis
☒	☐ Multivariate modeling or predictive analysis
